# Effect of soy consumption on liver enzymes, lipid profile, anthropometry indices, and oxidative stress in patients with non-alcoholic fatty liver disease: A systematic review and meta-analysis of clinical trials

**DOI:** 10.22038/ijbms.2020.46854.10797

**Published:** 2020-10

**Authors:** Aida Zarei, Cristina Stasi, Marzieh Mahmoodi, Seyed Jalil Masoumi, Morteza Zare, Mohammad Jalali

**Affiliations:** 1 Student Research Committee, Shiraz University of Medical Sciences, Shiraz, Iran; 2 Nutrition Research Center, School of Nutrition and Food Sciences, Shiraz University of Medical Sciences, Shiraz, Iran; 3 Interdepartmental Hepatology Center MASVE, Department of Experimental and Clinical Medicine, Careggi University Hospital, Florence, Italy

**Keywords:** Lipid peroxidation, Malondialdehyde, Meta-analysis, Nonalcoholic fatty liver, Nonalcoholic steatohepatitis, Soy

## Abstract

The present systematic review and meta-analysis was conducted to investigate the effects of soy intake on liver enzymes, lipid profile, anthropometry indices, and oxidative stress in non-alcoholic fatty liver disease (NAFLD). A systematic search was undertaken in PubMed, Embase, Scopus, Web of Science, and Cochrane Library covering up to 10 January 2020. A fixed-effect or random-effects models were applied to pool mean difference (MD) and its 95 % confidence intervals (CI). Four clinical trials comprising 234 participants were included in the meta-analysis. Compared to the controls, alanine aminotransferase (ALT) levels (MD=-7.53, 95% CI=[-11.98, -3.08], *P*=0.001, I^2^=0.0 %), body weight (MD=-0.77, 95 % CI=[-1.38, -0.16], *P*=0.01, I^2^=36.9%), and the concentration of serum Malondialdehyde (MDA) (MD=-0.75, 95% CI=[-1.29, -0.21], *P*=0.007, I^2^=63.6%) were significantly changed following soy intake. Lipid profile was not significantly affected by soy intake. Moreover, no evidence of a significant publication bias was found. The present study suggests lowering effects for soy intake on ALT levels, body weight, and MDA in nonalcoholic liver patients. Therefore, further large-scale and well-designed clinical trials are needed to find conclusive findings.

## Introduction

The global prevalence of nonalcoholic fatty liver disease (NAFLD) in the general population is estimated at 24% (1). NAFLD consists of two pathologically distinct conditions: NAFLD and non-alcoholic steatohepatitis (NASH) (2). Histologically, NAFLD is defined by predominantly macrovesicular steatosis and the presence of visible steatosis in > 5% of hepatocytes (3). The more severe forms with both lobular and/or portal inflammation, ballooning hepatocyte injury typically with a predominantly centrilobular (acinar zone 3) distribution in adults, and absence or presence of fibrosis in differing patterns of distribution to the end stage of cirrhosis are generally accepted as NASH (4). Common conditions with established association with NAFLD are obesity, Type 2 diabetes mellitus (T2DM), dyslipidemia, and metabolic syndrome (5). Moreover, patients with NAFLD have an increased risk of structural and functional cardiovascular disease (6). According to the data of the World Health Organization, cardiovascular disease is the leading cause of death globally, taking an estimated 17.9 million lives each year. Subjects at risk of cardiovascular disease may have raised blood pressure, glucose, and lipids, be overweight and obese (7). 

EASL (European Association for the Study of the Liver) and AASLD (American Association for the Study of Liver Diseases) recommend ultrasound examination and liver enzyme determination for the diagnostic assessment and monitoring of disease severity in the presence of suspected NAFLD and metabolic risk factors (2).

Given the very high prevalence of NAFLD as well as obesity, the question of how to reduce the incidence of cardiovascular events with food lifestyles is extremely intriguing and relevant. Soybean is a legume that contains high-quality protein (~40%), polyunsaturated fatty acids (~18%), carbohydrates (~8%), and dietary fibers (~17%) (8). Soybean has cholesterol-lowering effects and potentially modulates gut microbiota, shifting the gut microbiota composition toward an increment in Lactobacilli, Bifidobacterium, and Firmicutes to Bacteroidetes ratio (9).

Several lines of evidence indicate that soybean consumption showed a wide range of bioactivities including antioxidative, hepatoprotective, and cardiovascular protective effects (10, 11). 

In NAFLD patients, fat accumulation in hepatocytes provides a potential substrate for lipid peroxidation and reactive oxygen species (ROS) toxicity. An excessive ROS production enhances lipid peroxidation that in turn leads to the formation of other reactive metabolites in the liver, such as 4- hydroxy-2-nonenal (4-HNE) and malondialdehyde (MDA)(12). MDA is one of the most important and reliable markers of oxidative stress in clinical situations (13).

Studies in animals demonstrated that isoflavones prevent NAFLD and adiposity through regulation of peroxisome proliferator-activated receptors (PPARs), fatty acid β-oxidation, and oxidative stress, and they reduce *de novo* hepatic lipogenesis via ChREBP signaling and fat mass via the activation of anti-adipogenic WNT signaling, inhibiting the release of steatotic, or steatohepatitic adipocytokines, such as TNFα and ghrelin (14). 

The study of Gudbrandsen *et al*. (2009) demonstrated that obese rats with liver steatosis treated with soy proteins showed improved liver inflammation biomarkers such as alanine aminotransferase (ALT), aspartate aminotransferase (AST), TNF-alpha, and IL-1 (15).

Moreover, some trials found positive effects of soy intake on NAFLD patients (16, 17), however, some others did not (18, 19). Based on these premises, this systematic review and meta-analysis was conducted to investigate the effects of soy intake on liver enzymes, lipid profile, anthropometry indices, and oxidative stress in patients with NAFLD.

## Materials and Methods

The present study was conducted according to the Preferred Reporting Items for Systematic reviews and Meta-Analyses (PRISMA) was used to demonstrate the process of study selection (20).

***Search strategy***

Two independent authors (MJ and AZ) in PubMed, Embase, Scopus, Web of Sciences, and Cochrane Library undertook an online systematic search to detect clinical trials investigating the effects of soy consumption on liver enzymes, lipid profile, anthropometry indices, and NAFLD from the first available time up to 10 January 2020 without any limitation in language, time, or type of study. For it, the following pattern was used for the systematic search: [Keywords for soy] AND [Keywords for subjects and disease]. Moreover, the lists of references and Google Scholar were hand-searched by the named authors to avoid missing any relevant study.

***Inclusion and exclusion criteria***

Studies with the following traits were selected for meta-analysis: (1) being clinical trials (parallel or cross-over), (2) reporting usable and sufficient data of at least one of the interested outcomes including liver enzymes (ALT or/and AST), lipid profile (TG, TC, HDL-C, LDL-C), anthropometry indices (BMI or/and body weight) or/and oxidative stress (MDA), (3) having human design, and (4) being English in language. In contrast, exclusion criteria were: (1) having complication of other metabolic diseases besides NAFLD or NASH, (2) reporting insufficient data about change of outcomes at the end of trial from baseline, (3) having less than 8 weeks intervention duration, and also (4) being gray literature including editorials, brief reports, conference abstracts, dissertations, patents or/and book chapters.

***Data collection and risk of bias appraisal***

Two independent authors (MJ and AZ) evaluated the identified records through a systematic search for eligibility. Given selecting final eligible articles, following data were extracted by MJ and AZ, independently: first authors’ last names, publication year, country, sample size, type of intervention and control, duration of treatment, net changes of outcomes and their standard deviation (SD), mean age of participants in intervention group, and risk of bias of the included trials. Any doubts were resolved through discussion between MJ and AZ. Also, MZ was asked to double-check all of data. Joanna Briggs Institute (JBI) approach was used to assess the risk of bias (21).

***Statistical analysis***

Given statistical heterogeneity between studies, fixed-effect, or random-effects models were done to pool all data and calculate the mean difference (MD) and its 95% confidence intervals (CI) (22). To assess the statistical heterogeneity, I^2^>50% (high) and *P*-value<0.05 (significant) were used (23). To calculate net change and SD of outcomes, following formulas were planned, respectively: [mean post-treatment – mean pre-treatment], SD= square root [((SD pre)^2^ + (SD post)^2^) – (2r × SD pre × SD post))]. Also, if IQR was reported instead of SD, SD for changes was obtained using the method proposed by Hozo* et al.* (24). Sensitivity analysis was planned to assess the impact of the individual study on the pooled result. A *P*-value of less than 0.05 was considered statistically significant. All data were analyzed using Stata 13.0.

## Results

***Literature search***

**Figure 1:**
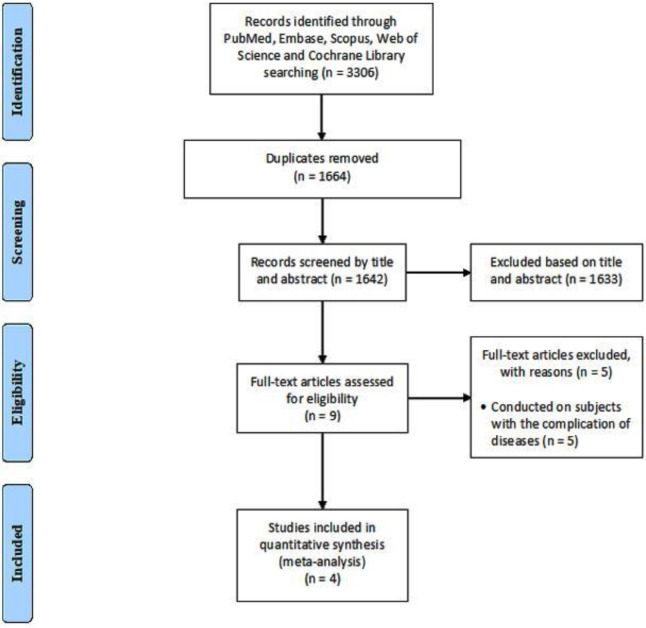
Flow diagram of the study selection process

Figure 1 shows the process of study selection. Briefly, 3306 references were recorded by the primary search. Of these, 1664 records were omitted through duplicates finding. Then, 1642 papers remained for title and abstract assessment, and 1633 of them were excluded. At the next step, 9 works of literature were assessed for eligibility and finally, 4 full-text articles were included in the meta-analysis (16-18, 25).

***Trial characteristics***

Table 1 outlines the demographic characteristics of the four included trials comprising 234 subjects. The timeframe of publication date was between 2014 and 2019. Moreover, all of the trials were conducted in Iran (16-18, 25). The mean age of participants in the treatment group ranged between 44.22 to 48.5 years old. Also, the intervention duration of included studies was 8 weeks (16-18, 25).

***Meta-analysis***

*The effect of soy on liver enzymes*

**Figure 2.**
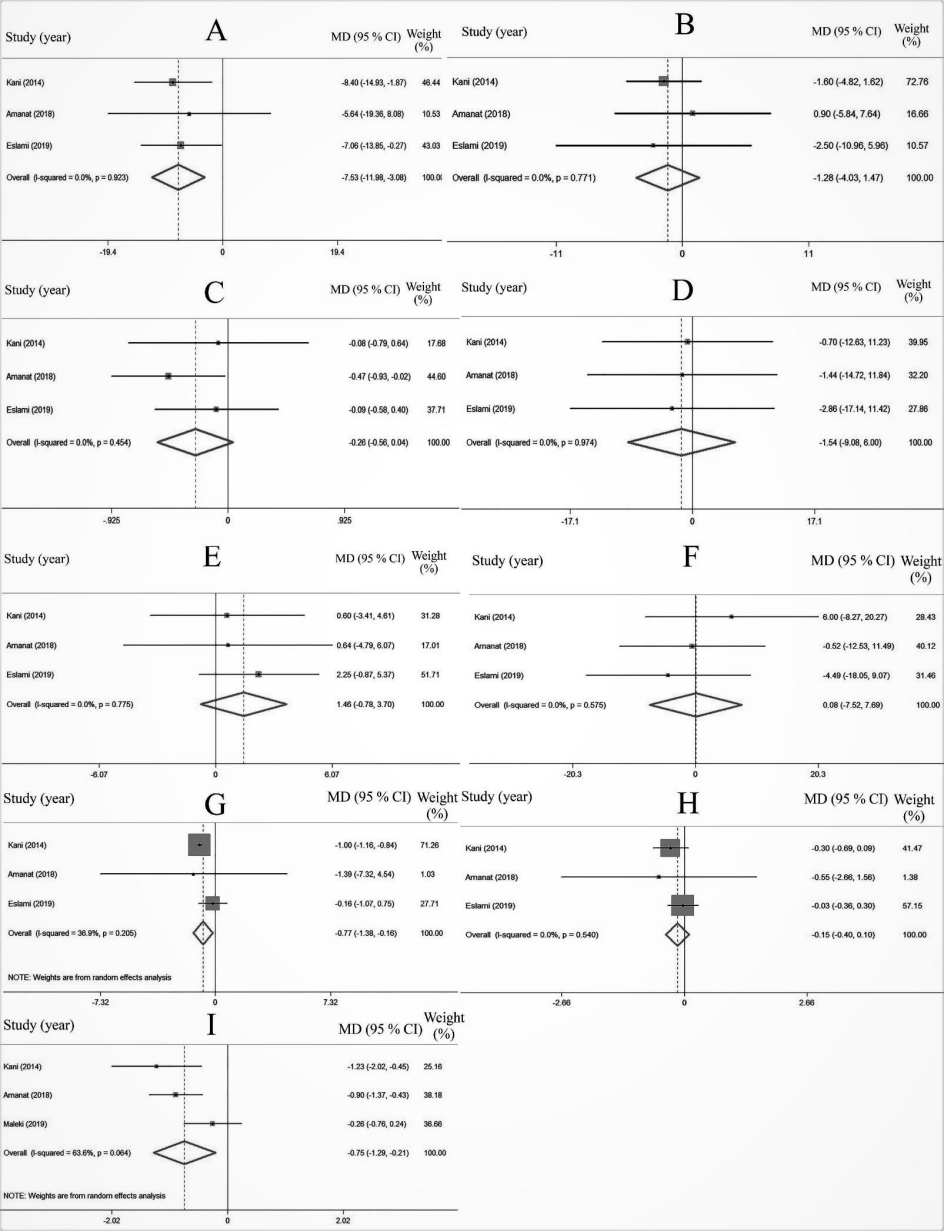
The effects of soy intake on ALT (A), AST (B), TG (C), TC (D), HDL-C (E), LDL-C (F), body weight (G), BMI (H), and MDA (I)

However, pooled estimates of 3 effect sizes (Figures 2A, B) revealed statistically and clinically significant effects of soy consumption on the concentration of serum ALT (MD=-7.53, 95% CI=[-11.98, -3.08], *P*=0.001, I^2^=0.0%); AST (MD=-1.28, 95% CI=[-4.03, 1.47], *P*=0.36, I^2^=0.0%) levels were not significantly affected as compared with the control subjects.

*The effect of soy on lipid profile*

Pooled estimates of 3 studies (Figure 2) reported non-significant effects of soy intake on serum TG levels (MD=-0.26, 95% CI=[-0.56, 0.04], *P*=0.91, I^2^=0.0%), TC (MD=-1.54, 95% CI=[-9.06, 6.00], *P*=0.68, I^2^=0.0%), HDL-C (MD=1.46, 95% CI=[-0.78, 3.70], *P*=0.20, I^2^=0.0%), and LDL-C (MD=0.08, 95% CI=[-7.52, 7.69], *P*=0.98, I^2^=0.0%) in comparison with the controls.

*The effect of soy on anthropometry indices*

Although, soy intake had a significant effect on the reduction of body weight (MD=-0.77, 95% CI=[-1.38, -0.16], *P*=0.01, I^2^=36.9 %), amount of body mass index (BMI) showed no statistically significant change (MD=0.15, 95% CI=[-0.40, 0.10], *P*=0.23, I^2^=0.0 %), compared with the controls (Figures 2G, H).

*The effect of soy on oxidative stress*

A forest plot of 3 datasets (Figure 2I) showed a significant reduction of MDA levels in comparison with the controls (MD=-0.75, 95% CI=[-1.29, -0.21], *P*=0.007, I^2^=63.6 %).

***Meta-regression***

Meta-regression was performed to investigate the potential sources of heterogeneity in serum MDA by mean age and publication year. Moreover, tau^2 ^was 4.75 in 3 included studies. As indicated in Table 2, meta-regression showed that the mean age of participants and publication year were not the sources of statistical heterogeneity.

***Publication bias and sensitivity analysis***

No evidence of significant publication bias was found in the meta-analyses (*P* for ALT=0.15, body weight=0.25 and MDA=0.66). Based on sensitivity analysis, none of the results was changed by removing an individual study at a time. 

**Table 1:** Demographic characteristics of the included studies in the meta-analysis of the effect of soy intake on NAFLD patients

**First author**	**Publication year**	**Country**	**Mean age**	**Sample size**	**Duration**	**Intervention**	**Control**
**Kani**	2014	Iran	48.5	15 / 15	8 weeks	Low-Calorie, low-carbohydrate soy-containing	Low-calorie, low-carbohydrate
**Amanat**	2018	Iran	44.22	41 / 37	8 weeks	Genistein	Placebo
**Eslami**	2019	Iran	46.25	32 / 32	8 weeks	Soy milk	Consumption not soy-based products
**Maleki**	2019	Iran	46.16	31 / 31	8 weeks	Soy milk	Consumption not soy-based products

**Table 2:** The results of the meta-regression of serum MDA by mean age and publication year for NAFLD patients

Covariates	Coefficient	*P*-value	95 % CI	Tau^2^
Mean age	-0.97	0.21	-5.23, 3.29	0.92
Publication year	0.78	0.22	-2.81, 4.39	1

ALT: aminotransferase; AST: aspartate aminotransferase; TG: triglycerides; TC: total cholesterol; HDL-C: high density lipoprotein cholesterol; LDL-C: low density lipoprotein cholesterol; BMI: body mass index; MDA: Malondialdehyde

## Discussion

To the best of our knowledge, the present systematic review and meta-analysis is the first study that evaluated the effect of soy consumption on liver enzymes, lipid profile, anthropometry indices, and oxidative stress in patients with NAFLD.

The meta-analysis of data from four clinical trials indicates significant effect of soy consumption on body weight, plasma ALT, and MDA level.

It seems that soy consumption had an important role in reversing NAFLD related complications via its anti-obesity and anti-oxidant properties.

Several studies have shown that the beneficial properties of soy are associated with isoflavones that may affect body metabolism and energy homeostasis through their interactions with estrogen receptors and exhibit estrogenic activity (26). In this perspective, it has been reported that isoflavones lead to reductions in the accumulation of lipids and the distribution of adipose tissue and thereby cause reduced body weight (27-29). According to these findings, our results demonstrate significant reduction of body weight, although a significant effect of soy intake on lipid profile has not been demonstrated. Due to the lack of specific pharmacological treatment for NAFLD patients, which complicates the management of these patients, Clinical Practice Guidelines for the management of NAFLD recommends in overweight/obese patients. A 7–10% weight loss as the target of most lifestyle interventions (30) in 27 obese people demonstrated that weight gain-induced steatosis, increasing the intrahepatic *de novo* lipogenesis from carbohydrates associated with a decrease in the elimination of fatty acids by intrahepatic fatty acid oxidation.

Liu *et al*. found that soy isoflavones decrease fat deposits in the liver through reducing adipogenesis and lipogenesis and activating the expression of PPAR-α to potentiate fatty acid oxidation in the liver (31). Hence, soy isoflavones could ameliorate the progression of NAFLD via decreasing ALT and improving liver structure (31), which is consistent with our findings.

Soy isoflavones also had antioxidant properties via scavenging free radicals and promoting the activity of antioxidant enzymes and thereby leading to protection against oxidative stress damage. In this view, Ibrahim *et al.* showed that administration of soy isoflavones decreases hepatic MDA and increases hepatic antioxidant enzymes (32). These results also are similar to our findings.

This study had several limitations. One of the important limitations is related to the small number of clinical trials with small sample sizes included in the analysis. Beside unhealthy lifestyle habits, genetic predisposition is another one risk factor for developing NAFLD, therefore another limitation of the study is that all trials included in the analysis are Iranians.

## Conclusion

Despite the limited number of studies and the limits of systematic review and meta-analysis, the results suggest lowering effect of soy intake on ALT levels, body weight, and MDA in NAFLD patients. Therefore, further large-scale and well-designed clinical trials are warranted to find conclusive findings.
